# High Accuracy Ultrasound Micro-Distance Measurements with PMUTs under Liquid Operation

**DOI:** 10.3390/s21134524

**Published:** 2021-07-01

**Authors:** Iván Zamora, Eyglis Ledesma, Arantxa Uranga, Núria Barniol

**Affiliations:** Departament d’Enginyeria Electrònica, Universitat Autónoma de Barcelona, 08193 Bellaterra, Spain; ivan.zamora@uab.es (I.Z.); eyglis.ledesma@uab.es (E.L.); arantxa.uranga@uab.es (A.U.)

**Keywords:** immersed distance measurement, multi-frequency continuous waves, phase measurement, PMUT, time-of-flight, ultrasound

## Abstract

Ultrasonic systems driven by multi-frequency continuous waves (MFCW) have been used for range distance measurement, offering high accuracy in long and medium range distance estimation. However, the use of continuous waves in very short-distance measurements causes large errors due to multipath reflections. This paper presents a new strategy to estimate very short relative distances with high accuracy based on the use of multi-frequency pulsed waves (MFPW). The proposed strategy allows to avoid the multipath reflections that appear when continuous waves are used, and it improves the achieved accuracy compared to the original MFCW method. To validate it, an 80 µm square AlScN piezoelectric micromachined ultrasonic transducer (PMUT) was chosen as a transmitter while a hydrophone was utilized as a target and receiver, immersed in fluorinert (FC-70) as a propagation medium. Three independent and consecutive tone-burst signals were transmitted successively. The selected frequencies are f1 = 2.3962 MHz, f2 = 2.327 MHz and f3 = 2.1195 MHz, giving first and second-order resolutions of 6.88 and 0.79 µm/°, respectively. Experimental results show a ±6.2 μm measured range error in a range of 3.5 mm, and therefore it represents a good candidate for ultrasound micro-profilometer applications under liquid operation.

## 1. Introduction

Time of flight (ToF) estimation has been a widely employed procedure to compute distances, using various forms of energy, such as ultrasound, radio frequency and light. Several techniques have been utilized for ultrasonic distance measurement systems to estimate the ToF. The simplest one is the delay estimation based on a short signal [1,2], where an ultrasound pulse is transmitted to the surrounding medium, and the elapsed time between the outgoing signal and the detection of the reflected echo (ToF) is measured. The distance to the reflecting target (d) is directly proportional to the ToF and the speed of the sound in the propagation medium (c), according to (1):

(1) $$d\;=\;\frac{c\cdot \mathrm{ToF}}{2}$$

Most of the systems based on the pulse-echo method employ the ToF estimation, based on cross-correlation techniques [3,4,5,6]. ToF estimation is obtained through the cross-correlation function between the transmitted and received signals. This method although is very robust against disturbances, it requires a high-level of signal processing.

An alternative method to the short signal delay estimation is the use of frequency modulated continuous wave (FMCW) [7,8]. In these systems, the transmitter emits a frequency modulated continuous wave (CW), which is reflected at the target and is collected by the receiver. ToF is proportional to the difference in frequency between the transmitted and the received signals [7,8]. To avoid multipath reflections, it is usual to shorten the continuous wave generating a Chirp signal, where the transmission frequency changes as a function of time, and the transmission time is limited to a pulse with a T_sweep_ duration [9,10,11]. Chirp modulation and FMCW methods both achieve the same range accuracy, but the use of chirp signals allows to discriminate multi-target echoes. However, both FMCW and Chirp based methods require a certain bandwidth, which limits the chosen transducer.

Finally, the ToF can also be estimated using unmodulated CW signals. In this case, the ToF is estimated based on the phase shift. The phase shift between the continuous electrical excitation signal and the continuous received signal is measured and it is used to compute the distance. This technique is relatively insensitive to disturbances since phase data can be sampled for a significant number of wave periods and therefore, random noise tends to cancel [12]. The main disadvantage of this method is the limited measurement range, due to fact that phase shift can only be measured in the 0–360 degree interval and therefore, the resulting distance range is limited to a wavelength. This drawback is solved by implementing two-frequency continuous wave (TFCW) [13] or multi-frequency continuous wave (MFCW) [12,14] based algorithms, where the phase shift of two or more frequencies are compared, allowing to measure distances much greater than one wavelength.

Although CW based systems show a good performance in medium and large range, in short distance measurements (mm) they suffer from multipath reflections, that can cause large range errors [15].

**Figure 1:**
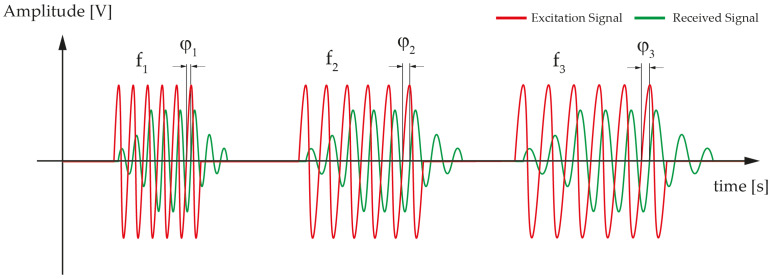
Excitation signals and received signals.

In this paper we present a new strategy that allows us to determine the relative position between a target and a reference point for short range measurements (mm) with high accuracy. It is based on the determination of the ToF using three burst-tone signals (each one composed of a fixed number of sinusoidal waves with a fixed frequency) that are sent sequentially, see Figure 1. The measurement of the phase shift using zero crossing along with a modified algorithm allows to discriminate short distances, avoiding distance dependent distortion due to multipath reflections.

As a proof of concept, we implemented a system that demonstrates the functionality of the proposed measurement strategy working in liquid for short distances measurement. The implemented system uses a piezoelectric micromachined ultrasonic transducer (PMUT) that works as an actuator and a commercial hydrophone that works as the target. The hydrophone position is lifted up in fixed steps and the distance between the hydrophone and a fixed reference point is measured.

The paper is organized as follows. Section 2 details the proposed measurement strategy. In Section 3 the experimental results for validation and comparison with other approaches are presented and conclusions are given in Section 4.

## 2. Method

The MFCW algorithm has been extensively applied in radio frequency distance measurement and in accurate air-coupled ultrasonic rangefinder [12]. It is based on the two frequency continuous wave (TFCW) method [13] where a third frequency is included in order to increase the maximum range without sacrificing accuracy.

The MFCW method consists of the generation of three different continuous ultrasound waves (f1, f2 and f3) that are transmitted sequentially from an ultrasound transducer. These three acoustic signals are produced by the application of the respective three electrical signals over the PMUT that works as a transducer. When the three ultrasound signals reach sequentially the target (in our case, the commercial hydrophone), three electrical signals are generated in response to the arrival of the ultrasound.

The phase shifts (φ_1_, φ_2_ and φ_3_) between the electrical received signal and the electrical excitation signal are measured, corresponding each one to each continuous wave (f_1_, f_2_ and f_3_). The distance d between the transducer and the target is computed as Equation (2) [12,14]:

(2) $$d\;=\;\mathrm{Int}[\frac{\Delta \varphi_{1,2}}{360}\cdot \frac{\Delta f_{1,3}}{\Delta f_{1,2}}]\cdot \frac{c}{\Delta f_{1,3}}\;+\;\mathrm{Int}[\frac{\Delta \varphi_{1,3}}{360}\cdot \frac{f_{1}}{\Delta f_{1,3}}]\cdot \frac{c}{f_{1}}\;+\;\frac{\varphi_{1}}{360}\cdot \frac{c}{f_{1}}$$

where Int [] is the integer operation, Δφ_1,2_ and Δφ_1,3_ are the phase differences (in degrees) between φ_1_, φ_2_ and φ_1_, φ_3_, respectively, Δf_1,2_ and Δf_1,3_ are the frequency differences between f_1_, f_2_ and f_1_, f_3_, respectively. The maximum allowed measured distance using the MFCW method is determined by c/Δf_1,2_. According to Equation (2), the ranging distance d is computed in three steps. The first one defines the largest resolution scale (c/Δf_1,3_)/360°, dividing the maximum range into Δf_1,3_/Δf_1,2_ divisions. In the second step, each division of the first step is subdivided into f_1_/Δf_1,3_ divisions, giving a finer resolution. In the final step, the phase shift φ_1_ is used to yield the highest-level resolution, defined as (c/f_1_)/360°. Considering this, the selection of the operation frequencies must achieve a trade-off between signal-to-noise ratio (SNR), resolution, and maximum measurable range. Therefore, f_1_, f_2_ and f_3_ must be within −3 dB PMUT bandwidth to maximize the SNR, f_1_ must be high enough, and f_3_ must be as far away as possible from f_1_ to maximize both, the first and second order resolutions. On the other hand, f_2_ must be close to f_1_ in order to increase the maximum allowed measured distance.

In our application, this algorithm shows some drawbacks. On the one hand and considering that the transducer will be relatively close to the target (hydrophone), the use of a continuous ultrasound wave causes the appearance of standing waves between the surface of the transducer and the target (multipath reflections). Multipath reflections of the acoustic waves produce an error in the phase shift measurements, leading to a non-linear distortion of the phase–distance relationship and therefore directly affecting the measurement results [15]. On the other hand, the use of the integer operation (Int []) in Equation (2) can generate errors of ±c/∆f_1,3_ (Equation (2) first term) and ±c/f_1_ (Equation (2) second term) in the distance estimation when Δφ_1,2_ gets closer to multiples of (360 × Δf_1,2_/Δf_1,3_) and Δφ_1,3_ gets closer to multiples of (360 × Δf_1,3_/f_1_), respectively.

Taking into account the multipath reflections drawback, some solutions have been proposed in [15] to reduce the non-linear distortion of the phase–distance relationship, but at the expense of increasing the overall measurement time and the computational complexity. In this work, we propose the use of multi-frequency pulsed waves (MFPW), where the three continuous waves are replaced by three burst signals, each one composed of a fixed number of sinusoidal signals with a fixed frequency (f1, f2 and f3, respectively). The number of cycles is determined limiting the transmission time to be smaller than the arrival time of the echo signal (the echo is generated by the reflection of the ultrasound signal when it reaches the target), see Figure 1 where a brief schematic of the applied signals is shown. This signal goes back to the transducer where it reflects again and reaches the target. Therefore, the transmission time is determined by the position of the target along with the time for the transducer to reach its steady state.

**Figure 2:**
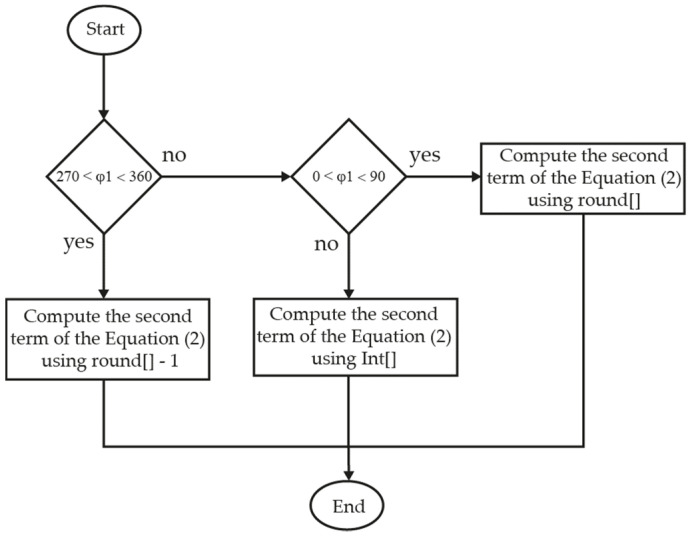
Algorithm flowchart used to compute the second term of Equation (2).

To enhance accuracy at points that generate Δφ_1,3_ phase differences multiples of 360 × Δf_1,3_/f_1_, which corresponds to distances multiples of λ_1_ (c/f_1_), we propose to apply on Equation (2) the round operation (Round []) on points located in the neighborhood of integer multiples of λ_1_, and to apply the integer Int [] operation on the rest of the points. To identify the neighborhood of integer multiples of λ_1_, the measured phase (φ_1_) is compared with ±90°. The proposed algorithm is described in Figure 2. If distances larger than c/∆f_1,3_ need to be measured, the same algorithm should be applied to the first term of Equation (2), using ∆φ_1,3_ instead of φ_1_ as a test variable.

## 3. Experimental Results and Discussion

To validate the proposed MFPW algorithm for ultrasound distance measurements in liquid, an 80 µm squared piezoelectric micromachined ultrasonic transducer (PMUT), fabricated with the MEMS-on-CMOS process from Silterra was used [16,17]. In this case the PMUT is composed of 1.2 μm AlScN (Aluminium Nitride doped with 9.5 % Scandium) sandwiched between aluminum electrodes and covered by 1.5 μm Silicon Nitride as elastic layer [18]. The PMUT was wire bonded to a printed circuit board (PCB) and immersed in Fluorinert (FC-70). A Signal Generator (Keysight 81150A, Santa Rosa, CA, USA) was used to drive the PMUT. The acoustic characterization of the PMUT in FC-70 (685 m/s) gives a resonance frequency for the first flexural mode of 2.327 MHz, and a −3 dB bandwidth of 740 kHz.

**Figure 3:**
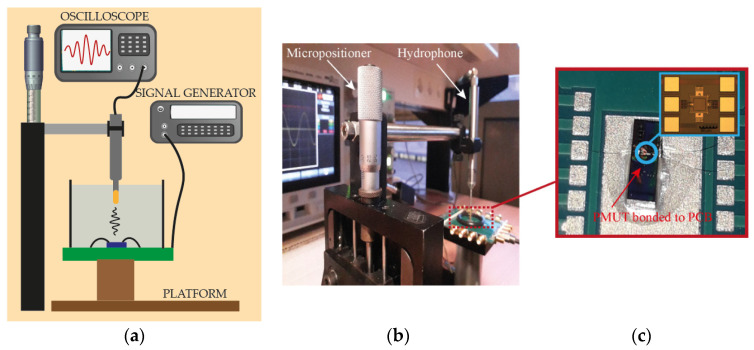
(**a**) The set-up diagram; (**b**) experimental set-up; (**c**) optical image of the PMUT device.

Figure 3 shows the experimental set-up used to perform the distance measurement experiments along with an optical image of the PMUT. It is composed of the designed PMUT (used as an actuator) and a commercial hydrophone (ONDA-HNC-1500, Sunnyvale, CA, USA) that was used both as a receiver and as a target (to measure the distance between the hydrophone and a fixed reference point). The commercial hydrophone was placed in a xyz-micro-positioner stage and its position was changed vertically in steps of 20 µm with 1 µm resolution (see Figure 3). The micro-positioner was used to validate the relative measured distances. The phase shift angle between the excitation signal and the amplified received signal was measured using the zero-crossing approach directly on the oscilloscope (Keysight DSOX3054A, Santa Rosa, CA, USA).

In order to achieve a trade-off between signal-to-noise ratio, maximum range and accuracy, three frequencies were chosen: f_1_ = 2.3962 MHz, f_2_ = 2.327 MHz and f_3_ = 2.1195 MHz, resulting in a 6.88 µm/° coarse-resolution and 0.79 µm/° fine-resolution.

Since we want to ensure that the echo-signals do not overlap in time with the measured signal, there is a minimum measurable range that can be computed using Equation (1), the sound velocity c = 685 m/s (previously calibrated) and the minimum unidirectional ToF. The minimum ToF is achieved when the f_3_ excitation signal and the start of the steady state of the received signal overlap in time, keeping in mind that this time has to be small enough to avoid the arrival of the first echo. Since our PMUT reaches its steady state at least after 4 cycles (4T, being T the period of the excitation signal), these two conditions force that the minimum time needed for the ultrasound signal to reach the target should be larger than two cycles (2T). Therefore, a minimum transmission time of six signal cycles is obtained. According to Equation (1), the minimum distance that assures that overlapping does not occur is around 750 μm.

To demonstrate the advantages of our MFPW strategy we performed two different experiments. The first one is aimed at comparing the performance of the MFPW versus the MFCW (Continuous Waves), while the second experiment analyzes the improvement in terms of absolute error and standard deviation when the proposed computation algorithm is used with the MFPW method.

### 3.1. Multi-Frequency Continuous Waves (MFCW) vs. Multi-Frequency Pulsed Waves (MFPW)

A first experiment was done to compare the use of continuous waves versus pulsed waves. The hydrophone was located 2.5 mm away from the transducer. To corroborate the distance, the PMUT was driven by four sine cycles at 2.327 MHz with 22 Vpp and the ToF was roughly estimated using an oscilloscope, based on the time the ultrasound signal needs to reach the hydrophone and the ultrasound speed in fluorinert.

After corroborating the position of the hydrophone, three excitation signals, each one with frequencies f_1_ = 2.3962 MHz, f_2_ = 2.327 MHz, and f_3_ = 2.1195 MHz, composed of a 16 period-long sine wave with an amplitude of 22 Vpp were applied consecutively. The micro-positioner was moved away axially using a micro-meter for 50 steps of 20 µm each one, giving a measurement range from 2.5 to 3.5 mm. The number of cycles was determined to assure the overlap of the excitation and the received signals, avoiding the overlap with multipath reflection signals.

Three phase shift angles φ_1_, φ_2_ and φ_3_ between the electrical received signal and the electrical excitation signal (one for each frequency) were measured at each distance point, and they were used as input data for the implemented algorithm. The number of cycles were increased by one each time the hydrophone was lifted one λ_1_.

**Figure 4:**
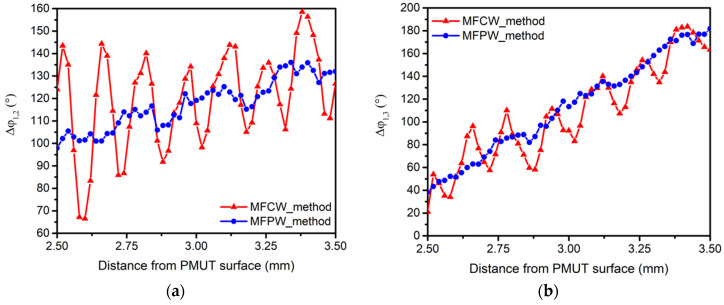
Measured phase difference using multi-frequency continuous wave (MFCW-red) and multi-frequency pulsed wave (MFPW-blue): (**a**) Δφ_1,2_; (**b**) Δφ_1,3_.

Figure 4 shows the measured phase differences, Δφ_1,2_ (Figure 4a) and Δφ_1,3_ (Figure 4b), when MFCW (red curves) and MFPW (blue curves) were used. The measured phase differences for the MFCW curves show a non-linear behavior with the distance, with a periodic error around 140 and 160 μm for Δφ_1,2_ and Δφ_1,3_, respectively. According to [15], this periodic component is given by the multipath reflections and it has a periodicity of λ_avg_/2 (where λ_avg_ = c/((f_1_ + f_2_)/2) for Δφ_1,2_ and λ_avg_ = c/((f_1_ + f_3_)/2) for Δφ_1,3_), which is in agreement with the obtained values. The blue curves, corresponding to the MFPW method, show a great improvement of the linearity, where it can be seen that the periodic error was suppressed. However, a residual distortion is still present, being mainly caused by the electronic noise of hydrophone’s pre-amplifier, which is explained later.

**Figure 5:**
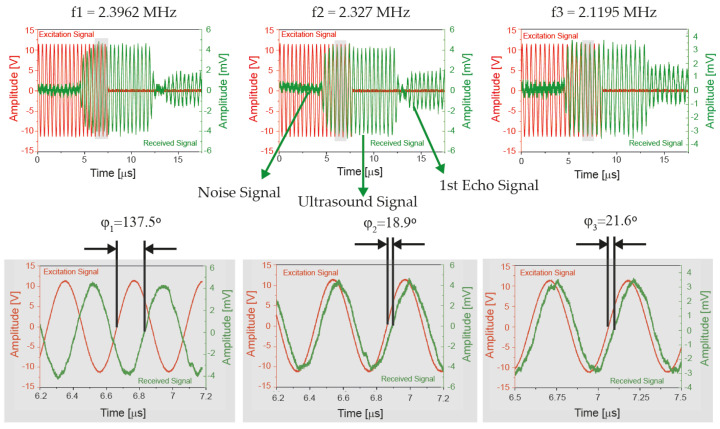
Measurement results 3 mm from PMUT surface. The red curves are the electrical excitation signals, and the green curves are the electrical received signals by the hydrophone.

Figure 5 shows the received signals by the hydrophone when it was placed 3 mm from the PMUT surface. As it can be seen the first echo arrives after the phase shift measurement is done, avoiding the non-linear distortion due to multipath reflections.

### 3.2. Relative Distance Measurement

Our main goal is to prove that the proposed strategy allows us to implement a highly accurate relative distance measurement. For this purpose, we fixed the reference point 2.5 mm away from the transducer and we measured the φ_1__2.5 mm, φ_2__2.5 mm and φ_3__2.5 mm phase angles at that distance.

Relative distances from this reference point were measured using the proposed new strategy. Again, the micro-positioner was moved away vertically for 50 steps of 20 µm each one, giving a measurement range from 2.5 to 3.5 mm that corresponds to a relative movement of 1 mm from the reference point.

**Figure 6:**
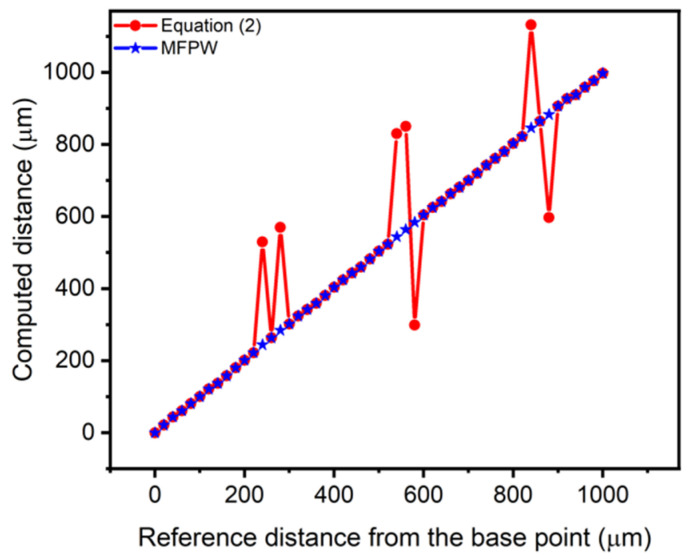
Experimental computed relative distances vs. real relative distances from the reference point (2.5 mm) using Equation (2) in red and with the proposed MFPW algorithm in blue.

To compute relative measurements, φ_1__2.5 mm, φ_2__2.5 mm and φ_3__2.5 mm were subtracted from each new phase angle measured on each new point. Figure 6 shows a graph of the real relative distance from the reference point (obtained according to the micro-positioner) versus the computed measured distance, when Equation (2) is used with the new algorithm. In particular, red points correspond to the use of the Int [] operator in Equation (2) while blue points are obtained using the proposed algorithm (Figure 2).

The use of Equation (2) (red points in Figure 6) shows that higher errors in the computed distance are obtained (around ±λ_1_) compared to the use of the new algorithm, when the measured distances are very close to an integer of λ_1_. Blue dots in Figure 6 correspond to the proposed MFPW strategy. It is proven that the jumps around integer multiples of λ_1_ are minimized thanks to the use of the proposed algorithm, as it was expected.

**Figure 7:**
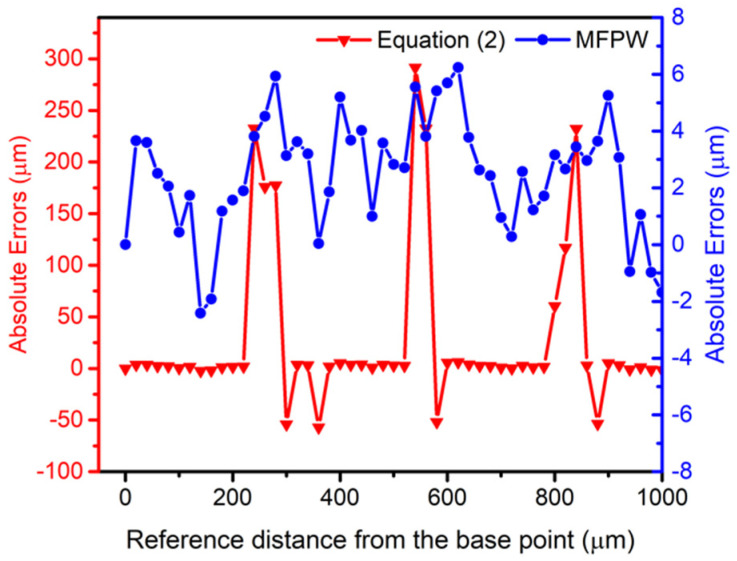
Obtained absolute errors using Equation (2) in red and the proposed MFPW algorithm in blue.

Figure 7 presents the obtained absolute distance errors computed as the difference between the average of 5 measurements done on each point and the real distance. In particular, a maximum error of ~291 µm is achieved using the Int [] operator while the maximum error is reduced to ~6.2 µm with the new MFPW strategy.

### 3.3. Uncertainty Analysis

To estimate the random errors, three uncertainty sources were identified: ultrasound velocity, oscillator stability and phase shift measurements at the zero crossing. Considering that the propagation medium used in our experiment (fluorinert FC-70, Sigma-Aldrich, St. Louis, MO, USA) is thermally and chemically stable [19], we assumed that the uncertainty due to the ultrasound velocity is negligible. On the other hand, taking into account that the used signal source in our experiment is a high stable oscillator (Keysight 81150A Signal Generator), the distance error caused by oscillator error will not be a concern. Therefore, the dominant random error source is the phase error. From [20], the standard deviation of the phase angle (σ_φ_ in radians) at zero crossing for a sinusoidal pulse is given by:

(3) $$\sigma_{\varphi }\;=\;\frac{1}{\sqrt{E/N_{0}}}$$

where E is the pulse energy and N_0_ is the power noise spectral density. If we consider the root mean squared (rms) values of both signal and noise, Equation (3) can be rewritten as:

(4) $$\sigma_{\varphi }\;=\;\frac{1}{V_{a,\mathrm{rms}}/\sigma_{n}}\;=\;\frac{1}{\mathrm{SNR}}$$

where V_a,rms_, σ_n_ are the rms values of both the signal and noise and SNR is the signal-to-noise ratio. Taking into account that the derivate of both, the integer and round functions are zero when their arguments belong to Real Domain, the standard deviation of the distance (σ_d_) according to Equation (2) can be calculated as:

(5) $$\sigma_{d}\;=\;\sqrt{{\left(\frac{\partial d}{\partial_{\varphi_{1}}}\sigma_{\varphi_{1}}\right)}^{2}}\;=\;\frac{c}{2\pi f_{1}}\cdot \frac{1}{\mathrm{SNR}}$$

Considering that the acoustic pressure distribution in far-field of the used PMUT presents an exponential dependence with the distance (2.66/z Pa_pp_) [18], being z the axial distance, the rms voltage at the output of Hydrophone’s preamplifier (V_a,rms_) is given by:

(6) $$V_{a,\mathrm{rms}}(z)\;=\;M\cdot \frac{2.66}{z}\cdot \frac{1}{2\sqrt{2}}$$

where M is the reception sensitivity at the output of hydrophone’s preamplifier (11.22 μV/Pa) [21]. In our case, the measured amplitudes are inside a range from 4.2 (corresponding to the reference point located at 2.5 mm) to 3 mV_rms_ (3.5 mm). Combining Equations (5) and (6), the standard deviation of the distance has a linear dependency with the distance, given by:

(7) $$\sigma_{d}(z)\;=\;\frac{c\sigma_{n}}{10.53\cdot 10^{-6}\cdot 2\pi f_{1}}\cdot z$$

**Figure 8:**
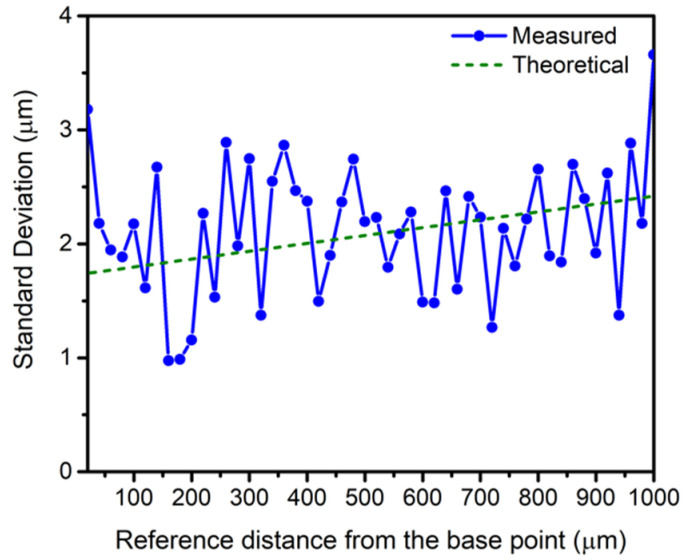
Experimental and theoretical σ_d_ vs. relative distances from the reference point (2.5 mm).

Considering the chosen frequencies, Equation (7) can only be used from the minimal measurable distance (750 μm) to the maximum range (c/Δf_1_ = 9.8 mm). Figure 8 shows the measured standard deviation for each distance point, and the theoretical standard deviation obtained from Equation (7), considering that the output-referred noise of the preamplifier (AH-2010 from Onda) is 160 μVrms in its 3-dB bandwidth (from 50 kHz to 25 MHz) [21].

Considering that a small number of measurements were performed (5 measurements), calculation of the confidence interval was done assuming that our measurements have a Student’s t distribution. Taking into account the maximum value of the standard deviation (see Figure 8), the 70% confidence limit is ~1.95 μm.

Table 1 compares the proposed MFPW algorithm with other reported methods implemented to measure distances using ultrasound. In order to compare the performance of the different methods, it is necessary to take into account the two main parameters that influence the accuracy: the central frequency and the SNR (Equation (5)) although the measurement distances can be completely different. Considering the final accuracy, our MFPW approach gives a range error of ±6.2 μm which is the smallest one reported. If we compare it with other MHz systems operated also in a liquid environment [4,5], the proposed method is more accurate at the expense of a reduced measurement range. Consequently, the proposed MFPW provides an attractive alternative in liquid environment at high frequencies for target micro-positioning with fine steps. 

## 4. Conclusions

This work presents a new strategy applied to an ultrasound system that allows the measurements of relative distances with high accuracy, using a PMUT with a modified multi-frequency phase shift with three transmission frequencies. The new approach, MFPW, improves the linear dependence between phase angles and target’s range avoiding the multipath reflections. It improves the accuracy in relation to the traditional MFCW algorithm, decreasing considerably the error around integer multiples of the wavelength. The experimental verification was made using an 80 µm AlScN squared PMUT in liquid environment (FC-70), achieving a measured range error of ±6.2 μm in 3.5 mm, offering better performance than the described algorithm by Equation (2), under the same conditions. Integration of the presented AlScN PMUT over CMOS circuitry would derive SNR levels comparable with those reported here. Considering this, a very compact and low power ultrasound distance measurement system based on a single PMUT using the MFPW strategy will achieve very high accuracy for short distances in liquid environments.

## Figures and Tables

**Table 1:** Performance comparison of ultrasound measurement distance.

	[13] (2012)	[1] (2015)	[11] (2021)	[4] (2015)	[5] (2019)	This Work
Method	TFCW	Pulse-echo	Chirp modulation	Cross-correlation/PSM	Cross-correlation	MFPW
Transducer	N/A	AlN PMUT	PZT	Commercial (Vico WK-21B)	Commercial (Goworld 1P28)	AlScN PMUT
Propagation medium	Air	Air	Air	Liquid (distilled water)	Liquid (water)	Liquid (FC-70)
Frequency (kHz)	40/40.82	220	94–107	1000	1000	2392.6/2327/2119.5
SNR (dB)	N/A	33 ^2^ at 500 mm	25.26 ^3^ at 550 mm	N/A	N/A	25.5 at 3.5 mm
Standard deviation (μm)	155.7 ^1^ at 200 mm	N/A	N/A	40 at 200 mm	N/A	1.95 ^5^ at 3.5 mm
Range error (μm)	±136 at 200 mm	410_rms_ at 500 mm	18,700 ^4^ at 550 mm	N/A	±202 at 25 mm	±6.2 at 3.5 mm

^1^ Corresponds to the greater value of all reported. ^2^ Extracted from a SNR versus range graph. ^3^ Computed using the received power characterization in air of their transducer (−60 dBm) and the input-referred noise of the amplifier (12.2 μVrms). ^4^ Corresponds to the standard deviation of the absolute error. ^5^ Represents the 70% confidence limit considering that the measurements have a Student’s t distribution.

## Data Availability

Not applicable.
